# Predictive Modeling of Compressive Strength for Concrete at Super Early Age

**DOI:** 10.3390/ma15144914

**Published:** 2022-07-14

**Authors:** Xi Peng, Zhenxin Zhuang, Qiuwei Yang

**Affiliations:** 1 School of Civil and Transportation Engineering, Ningbo University of Technology, Ningbo 315211, China; pengxi@nbut.edu.cn; 2 Engineering Research Center of Industrial Construction in Civil Engineering of Zhejiang, Ningbo University of Technology, Ningbo 315211, China

**Keywords:** concrete, super early age, compressive strength, predictive model, regression analysis

## Abstract

The compressive strength of concrete is an important parameter in construction practice. At present, there are few reports on the prediction model of the compressive strength of concrete at a super early age. For some engineering vibration analyses, it is very necessary to study the growth law of compressive strength of concrete at a super early age. To this end, a new prediction model is proposed in this work to analyze the variation of compressive strength for the concrete at a super early age. The innovations of this work mainly lie in two aspects. The first innovation is to propose a new compressive strength-age mathematical model to predict the variation of compressive strength more accurately. The second innovation is to develop a new robust regression analysis method to obtain the fitting parameters in the mathematical model more effectively. Using the experimental data of the super early age concrete, the proposed prediction model is compared with the existing power function model and the hyperbolic function model. The results of the comparative study show that the prediction model proposed in this work is more reasonable and reliable. Taking C40 under natural curing as an example, it has been shown from the comparative study that: (1) The total fitting error of the proposed model is approximately 60% of that of the power function model, and approximately 17% of that of the hyperbolic model; (2) The fitting standard deviation of the proposed model is approximately 49% of that of the power function model, and approximately 15% of that of the hyperbolic model; (3) The 28 day strength of concrete predicted by the proposed model is more in line with the actual strength growth law of concrete.

## 1. Introduction

The mechanical properties of concrete at an early age are a key topic in the field of civil engineering [1,2,3]. On the one hand, the curing conditions of concrete can be optimized according to the development law of early-age mechanical properties. On the other hand, in order to study the influence of vibration load on concrete in some engineering practices, it is also necessary to obtain the relevant mechanical parameters of early-age concrete in advance. In recent decades, many scholars have carried out pieces of research on the mechanical properties of early-age concrete. Kim et al. [4] studied the concrete strength variation for different curing conditions with particular regard to the influences of curing time points with given temperatures. They found that the concrete with a high temperature at an early age attains a higher early-age strength but eventually attains a lower later-age strength. Gu et al. [5] investigated the concrete strength development by inspecting the harmonic response of the embedded piezoelectric sensor at early ages. It was found that the concrete strength increases at a fast rate during the first few days and at a decreasing rate after the first week. Lee et al. [6] presented a new model for estimating the setting and compressive strength of the early-age concrete based on the ultrasonic pulse velocity testing. The relationships among parameters in different concrete samples were found to be linear during the initial and final setting periods and parabolic after the final set at early ages. Yoon et al. [7] used the surface wave velocities to predict the concrete strength at an early stage. They proposed an evaluation formula for computing the early-age compressive strength of concrete with 95% prediction intervals. Oluokun et al. [8] found that the compressive strength and splitting tensile strength are related, and an increase in one generally is similarly reflected in an increase in the other. The tensile strength was found to be proportional to the 0.79 power of the cylinder compressive strength. From the experimental study, Yang et al. [9] found that the compressive strength development of high-strength concrete at an early age is related to the curing temperature histories. Benaicha et al. [10] investigated the strength evolution of four types of concrete such as self-compacting concrete, high-performance concrete, and high-performance synthetic or metallic fiber-reinforced concrete. They proposed a function to characterize the on-site strength after casting of concrete. Lee et al. [11] found that high-early-strength-concrete can reach approximately 50–70% of its design compressive strength in a day in ambient conditions. Based on regression analyses, they proposed a generalized rate-constant model to predict the compressive strength of HESC at an early age. Min et al. [12] presented some measures to develop early-stage strength for precast concrete of more than 10 MPa after 6 h of curing at room temperature without steam curing. Chen et al. [13] investigated the compressive and splitting tensile strengths of concretes cured for different periods and exposed to high temperatures. It was found that the early-age concrete cured after exposure to high temperatures up to 800 °C can regain 80% of the designed compressive strength. According to the conductivity at microwave frequency, Chung et al. [14] proposed a prediction model for the 28 day compressive strength of concrete. They found that the magnitude of the slope of strength–conductivity curves can indicate the grade of standard concrete during early ages. Voigt et al. [15] used the wave transmission and reflection measurements to obtain the microstructural changes during the setting and hardening process of the early-age concrete. They found that S-waves used in the transmission and reflection mode were sensitive to the inter-particle bonding caused by the cement hydration. Demirboga et al. [16] studied the relationship between ultrasound velocity and compressive strength of the early-age concrete. It was found that the relationship between ultrasound velocity and compressive strength was exponential. Velay-Lizancos et al. [17] studied the evolution of the kinetics of E-Modulus and its relationship with compressive strength of the early-age concrete. They found that the maturity correction for E-Modulus evolution, based on the activation energy of compressive strength, produced an accurate superposition the of E-modulus in the equivalent age domain. Jin et al. [18] used the maturity method to predict the compressive strength of early-age bisphenol F-type epoxy resin concrete. It was observed that the compressive strength of the bisphenol F-type epoxy resin concrete was heavily affected by the curing temperature. Oluokun et al. [19] studied the relative relationships between the elastic modulus, Poisson’s ratio, and the cylinder compressive strength of the early-age concrete. It was found that the elastic modulus is proportional to the 0.5 power of the cylinder compressive strength for the early-age concrete. Poisson’s ratio did not change appreciably with compressive strength development. Adesina et al. [20] found that the early age compressive strength of sodium carbonate-activated slag can be enhanced with the use of additives such as calcium oxide, calcium hydroxide, Portland cement, sodium hydroxide, and sodium silicate. Jiang et al. [21] investigated the effects of solid content, the binder dosage, the activator to binder ratio, the sodium silicate to sodium hydroxide ratio, and the curing temperature on mechanical properties of the early-age alkali-activated slag concrete. It is observed that the compressive strength increases with solid content and binder dosage, but at a decreasing rate. Kaszyńska [22] studied the relation between the amount and kinetics of heat generation and the early age compressive strength of high-performance concrete cured in the massive structure, where the temperature in concrete changes continuously. Burhan et al. [23] investigated the effect of three types of powder polymer (polycarboxylate superplasticizer) in terms of setting time of cement, workability, density, and compressive strength of concrete until 28 days of curing. It is observed that the polymers are more effective than silica fume in improving the workability and compressive strength of concrete. Voigt et al. [24] used the ultrasonic wave reflection method to evaluate the compressive strength development of portland cement mortars. They found that the relationship between reflection loss and compressive strength is independent of curing temperature. Najm et al. [25,26] studied the colour and strength change of the sustainable concrete containing waste ceramic and hybrid fibre in ambient and elevated temperatures. It was found that the appearance of colour alteration usually coincided with the onset of a significant strength loss due to elected temperatures. The artificial neural networks and multiple linear regressions were used in their work to predict the compressive and tensile strength of concrete. Gutsch [27] carried out the experimental research on the thermal and mechanical properties of early-age concrete and proposed the corresponding mathematical models to describe the laws of strength development. Wei et al. [28] carried out the systematic creep comparison for early age concrete under different loading types and curing conditions. Their research results help to gain confidence about the predicted stress and the strain when different creep models are used.

Although many achievements have been made in the research of mechanical properties of early-age concrete, it is still necessary to study a more accurate predictive model for concrete strength-age at the super early age. Generally, the measured strength data of concrete are often very discrete because concrete is a brittle material. It is also necessary to study more robust regression analysis methods to deal with these discrete data. In view of this, the innovations of this work mainly lies in two aspects. The first innovation is to propose an improved mathematical model to predict the variation of compressive strength with concrete age more accurately. The second innovation is to develop a new robust regression analysis method to obtain the fitting parameters more effectively. The proposed robust regression analysis method is especially suitable for analyzing the mechanical characteristics of materials with great discreteness such as concrete. Using the experimental data, it was found that the proposed new compressive strength-age model has a better prediction accuracy than the existing models. The proposed robust regression analysis method can effectively overcome the great discreteness in the measured data. The framework of this study is organized as follows. Section 2 briefly reviews two existing compressive strength-age models and then proposes a new mathematical model for the concrete at a super early age. Section 3 presents the traditional and new regression analysis methods for comparison. Section 4 presents the verification of the proposed methods by using the experimental data of the super early-age concrete. Finally, the conclusions of this work are summarized in Section 5.

## 2. Compressive Strength-Age Predictive Model

As stated before, it is very necessary to propose an effective compressive strength-age model to predict the compressive strength of concrete at all times for engineering application. In this section, two existing compressive strength-age models are briefly reviewed firstly and then a new compressive strength-age model is proposed. One of the commonly used strength-age models is the power function model [29,30] as

(1) $$c(t)=x_{3}t^{3}+x_{2}t^{2}+x_{1}t+x_{0}$$

where c(t) is the function of compressive strength with time, t is the time from the beginning of concrete mixing (unit: hour), $x_{0}$, $x_{1}$, $x_{2}$, and $x_{3}$ are the four fitting parameters. Generally, the power function model has a high accuracy for predicting the compressive strength of concrete before 7 days, but a poor accuracy for predicting the compressive strength after 7 days. In particular, the power function model can not be used to predict the compressive strength of concrete after 28 days.

Another commonly used strength-age model is the hyperbolic model [31,32] as

(2) $$c(t)=\frac{t}{x_{0}+x_{1}t}$$

where $x_{0}$ and $x_{1}$ are the two fitting parameters for the hyperbolic model. The advantage of the hyperbolic model is that the predicted compressive strength gradually approaches a fixed value with the growth of time, which conforms to the actual law that the concrete strength remains basically unchanged in the later stage. The disadvantage of the hyperbolic model is that the fitting accuracy is not satisfactory for some cases with very discrete data.

In view of the shortcomings of these two models, an improved mathematical model is proposed to predict the variation of compressive strength with concrete age as

(3) $$c(t)=\frac{t}{x_{0}+x_{1}t+x_{2}/e^{t}}$$

where $x_{0}$, $x_{1}$, and $x_{2}$ are the three fitting parameters for this new model. Comparing Equation (3) with (2), a term of $x_{2}/e^{t}$ is added to the new model to improve the fitting accuracy for the super early-age stage. Obviously, the new model also has the advantage of the hyperbolic model, that is, the predicted compressive strength tends to be a constant over time.

## 3. Regression Analysis Method

The above three prediction models are all nonlinear models. In order to calculate the fitting parameters, these nonlinear models are generally converted into linear regression models. The multiple linear regression is often used to predict the compressive and tensile strength of concrete [26]. Taking the power function model as an example, the linear regression equation for this model can be expressed as

(4) {y}=Π·{x}

(5) $$\left\{y\right\}=\left\{\begin{array}{c} c(t_{1})\\ \vdots \\ c(t_{n}) \end{array}\right\}$$

(6) $$\left\{x\right\}=\left\{\begin{array}{c} x_{0}\\ \vdots \\ x_{3} \end{array}\right\}$$

(7) $$\Pi =\left[\begin{array}{cccc} 1 & t_{1} & \cdots & t_{1}^{3}\\ \vdots & \vdots & \ddots & \vdots \\ 1 & t_{n} & \cdots & t_{n}^{3} \end{array}\right]$$

where $c(t_{n})$ is the measured compressive strength of time $t_{n}$, Π is the coefficient matrix of this linear regression equation. Note that Equation (4) is an overdetermined equation since the number of equations is greater than the number of unknowns. Traditionally, the unknown regression coefficients {x} can be calculated by the least square solution of Equation (4) as

(8) $${\left\{x\right\}}_{c}={\left(\Pi^{T}\Pi \right)}^{-1}\Pi^{T}\left\{y\right\}$$

where the superscript “*T*” denotes the matrix transposition. Similarly, the fitting parameters for the hyperbolic model and the proposed model can also be obtained by the above traditional regression analysis. However, the mechanical properties of concrete material are often very discrete. Therefore, the traditional regression method based on the least square solution may not be suitable for analyzing the mechanical parameters of brittle materials such as concrete. It is very necessary to develop a robust regression method that can effectively deal with highly discrete data. To this end, a new weighted regression analysis method is proposed in this work to deal with the discrete data in order to obtain more reliable fitting parameters. The core idea of the proposed robust regression analysis is to reduce the weight coefficient of the measured data with relatively large deviation. Firstly, the residual error is defined as

(9) $$\left\{v\right\}=\left|\Pi \cdot {\left\{x\right\}}_{c}-\left\{y\right\}\right|$$

where {v} is the residual error vector whose element is the absolute deviation between the measured data with the calculated data obtained by the least square solution of Equation (8). Then, the mean and standard deviation of {v} can be calculated to evaluate the relative deviation by

(10) $$r_{i}=\left|v_{i}-m_{v}\right|-3s_{v}$$

where $r_{i}$ is the i-th relative deviation, $v_{i}$ is the i-th element of {v}, and $m_{v}$ and $s_{v}$ are the mean and standard deviation of {v}, respectively. According to the calculated result of Equation (10), the diagonal matrix of weight coefficient can be obtained as

(11) $$W=\left[\begin{array}{cccc} w_{1} & 0 & 0 & 0\\ 0 & \ddots & 0 & 0\\ 0 & 0 & w_{i} & 0\\ 0 & 0 & 0 & \ddots \end{array}\right]$$

(12) $$w_{i}=\{\begin{array}{c} e^{\frac{v_{\min}}{v_{i}}-1},\;if\;r_{i}<0\\ 0\;\;\;\;\;,\;\;if\;r_{i}\geq 0 \end{array}$$

where $v_{\min}$ is the minimum value of {v}. With the weight coefficient matrix determined by the above Equations, the robust regression solution of the unknowns {x} can be obtained by

(13) $${\left\{x\right\}}_{r}={\left(\Pi^{T}W^{2}\Pi \right)}^{-1}\Pi^{T}W^{2}\left\{y\right\}$$

## 4. Experimental Data Verification

**Figure 1:**
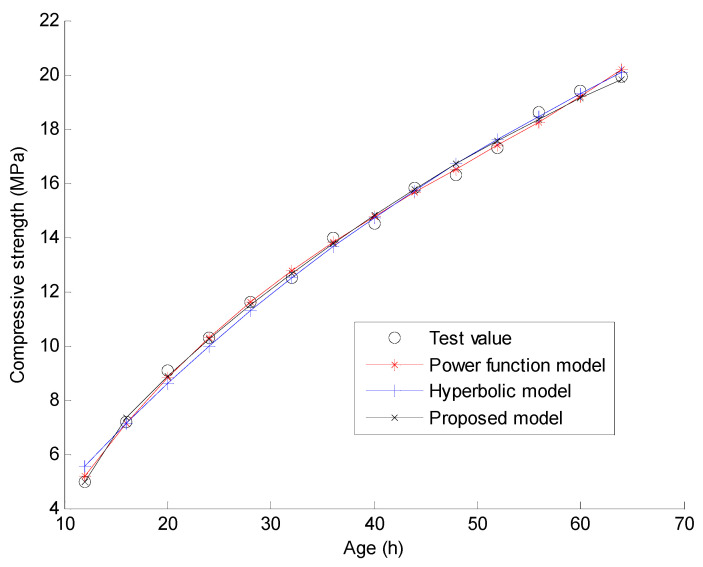
Curve fitting results using the three models for C30 under natural curing.

**Figure 2:**
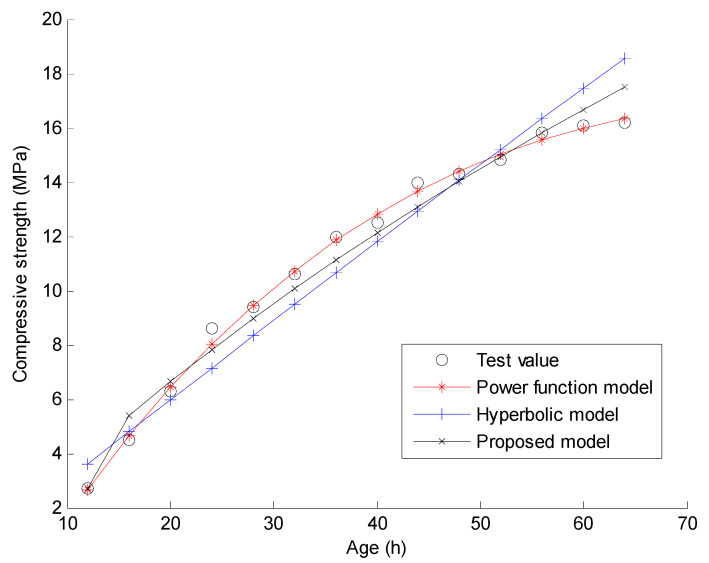
Curve fitting results using the three models for C30 under standard curing.

**Figure 3:**
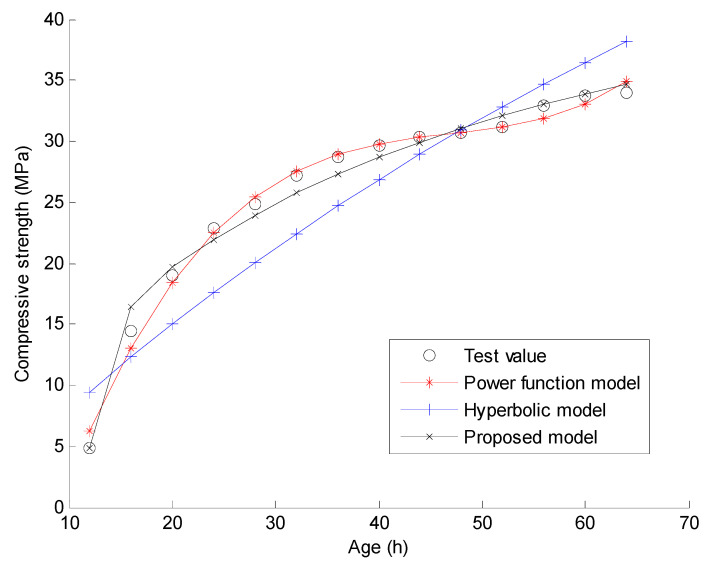
Curve fitting results using the three models for C40 under natural curing.

**Figure 4:**
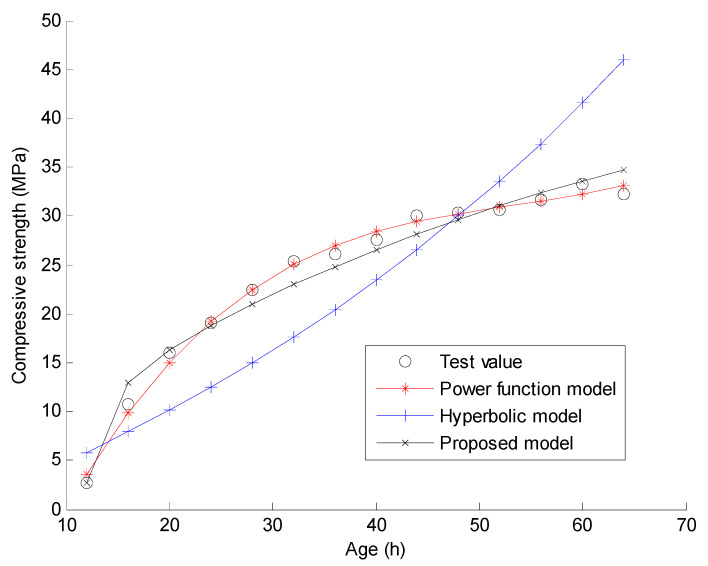
Curve fitting results using the three models for C40 under standard curing.

In this section, the experimental data conducted by Zhao in reference [33] are used to verify the proposed compressive strength-age model and the new regression analysis method. The main materials in the experiment include cement, fine aggregate, coarse aggregate, and water. Powdered naphthalene superplasticizer and fly ash are used in the process of mixing concrete. The measured compressive strength data of C30 and C40 concrete in the experiment are used for the verification of the proposed method. In the experiment, C30 and C40 indicate that the targeted strengths for the mix design are 30 MPa and 40 MPa, respectively. The quantities of the materials used for C30 concrete are: water 169 kg/m^3^, cement 357 kg/m^3^, fine aggregate 712 kg/m^3^, coarse aggregate 1115 kg/m^3^, water reducing agent 8.82 kg/m^3^, and fly ash 63 kg/m^3^. The quantities of the materials used for C40 concrete are: water 123 kg/m^3^, cement 357 kg/m^3^, fine aggregate 712 kg/m^3^, coarse aggregate 1115 kg/m^3^, water reducing agent 8.82 kg/m^3^, and fly ash 63 kg/m^3^. Reference [33] gives the measured compressive strengths of the 150 mm × 150 mm × 150 mm concrete cubes under natural curing and standard curing when the curing time t = 12, 16, 20, 24, 28, 32, 36, 40, 44, 48, 52, 56, 60, and 64 h, respectively. As shown in Figure 1, Figure 2, Figure 3 and Figure 4, these measured compressive strength data are marked by the symbol “o” for the four cases. In the experiment, the natural curing refers to curing concrete specimens wrapped with plastic film in the natural environment maintenance condition. The standard curing refers to curing in the special curing room with 20 ℃ and above 95% relative humidity. Using the proposed regression analysis method, Equations (14)–(16) present the specific curve equations by the three prediction models based on the measured compressive strength data for C30 under natural curing conditions. Equations (17)–(19) present the specific curve equations by the three prediction models for C30 under standard curing conditions. For C40 under natural curing, Equations (20)–(22) present the specific curve equations corresponding to the three prediction models. For C40 under standard curing, Equations (23)–(25) present the specific curve equations corresponding to the three prediction models.

(14) $$c(t)=0.0001t^{3}-0.0107t^{2}+0.7363t-2.2229$$

(15) $$c(t)=\frac{t}{1.9309+0.0197t}$$

(16) $$c(t)=\frac{t}{1.8233+0.0219t+5.1068\times 10^{4}/e^{t}}$$

(17) $$c(t)=0.0000t^{3}-0.0071t^{2}+0.6768t-4.4582$$

(18) $$c(t)=\frac{t}{3.2975+0.0024t}$$

(19) $$c(t)=\frac{t}{2.7063+0.0149t+2.5409\times 10^{5}/e^{t}}$$

(20) $$c(t)=0.0005t^{3}-0.0703t^{2}+3.3809t-25.0958$$

(21) $$c(t)=\frac{t}{1.1757+0.0079t}$$

(22) $$c(t)=\frac{t}{0.6401+0.0189t+2.5763\times 10^{5}/e^{t}}$$

(23) $$c(t)=0.0003t^{3}-0.0496t^{2}+2.7674t-22.9892$$

(24) $$c(t)=\frac{t}{2.2317-0.0131t}$$

(25) $$c(t)=\frac{t}{0.944+0.0141t+5.4236\times 10^{5}/e^{t}}$$

Furthermore, Figure 1, Figure 2, Figure 3 and Figure 4 present the comparisons of the test values and the calculated values by the three prediction models for the four cases. Note that the predicted value of the concrete compressive strength at any time is calculated by one of Equations (14)–(25). Taking the power function model as an example, the analytical value of compressive strength for C30 under 12 h natural curing can be easily calculated by Equation (14) with t = 12. When the predicted value at each time is calculated, the total fitting error of the compressive strength can be obtained as the square root of the sum for the squares of the difference between the predicted value and the test value at each time. Assuming γ(t) denotes the test value of the compressive strength for time t, the calculation formulas of the total fitting error ε are shown as Equations (26) and (27).

(26) $$\varepsilon =\sqrt{\sum_{t=12,16,\cdots ,64}\delta {\left(t\right)}^{2}}$$

(27) δ(t)=|c(t)−γ(t)|

Simultaneously, the calculation formulas of the fitting standard deviation σ are shown as Equations (28) and (29).

(28) $$\sigma =\sqrt{\frac{\sum_{t=12,16,\cdots ,64}{\left(\delta (t)-\bar{\delta }\right)}^{2}}{n}}$$

(29) $$\bar{\delta }=\frac{\sum_{t=12,16,\cdots ,64}\delta (t)}{n}$$

where n is the number of sample size. For t = 12, 16, …, 64 h, n = 14 is used in Equations (28) and (29) since there are 14 test time points in total. Using the above equations, Table 1 and Table 2 present the total fitting errors and standard deviations of the three prediction models for these four cases, respectively. Table 3 presents the predicted compressive strengths of concrete with 28 days by the three compressive strength-age models.

From Figure 1, Figure 2, Figure 3 and Figure 4, one can find that the curve shape corresponding to the proposed prediction model is more in line with the actual growth law of concrete strength. From Table 1 and Table 2, it is found that the total fitting error and standard deviation of the proposed prediction model are the smallest of the three models except for the case of C30 under standard curing. Taking C40 under natural curing as an example, the following quantitative conclusions can be drawn: (1) The total fitting error of the proposed model is approximately 60% of that of the power function model, and approximately 17% of that of the hyperbolic model; (2) The fitting standard deviation of the proposed model is approximately 49% of that of the power function model, and approximately 15% of that of the hyperbolic model. From Table 3, it can be seen that only the 28 day strength data predicted by the proposed compressive strength-age model are reliable. Note that only the test data of compressive strength before 3 days are used in this example. It can be expected that the proposed model will obtain more accurate prediction results if more test data after 3 days are used. The above results show that the proposed prediction model has a higher prediction accuracy and a wider application range than the existing models. In comparison, the total fitting error and standard deviation of the hyperbolic model are the largest of the three models. This indicates the instability of the hyperbolic model in fitting the strength data of concrete at a super early age. As a result, the 28 day strength data predicted by the hyperbolic model also fluctuate greatly. For the power function model, the total fitting error and standard deviation are acceptable, but the predicted 28 day strength data are unacceptable since these predicted values are much larger than expected. On the whole, only the proposed model has the advantages of a high fitting accuracy and a high prediction accuracy. These results can be further analyzed from a mathematical point of view as follows. (1) According to Equations (1)–(3), the number of the fitting parameters in the power function model, the proposed model, and the hyperbolic model are 4, 3, and 2, respectively. Generally, the less the number of fitting parameters, the worse the fitting accuracy. This may be the mathematical essential reason for the worst fitting accuracy of the hyperbolic model. (2) From Equation (1), it is obvious that the predicted compressive strength by the power function model increases with time if $x_{3}$ > 0. This violates the real law of concrete strength development. Thus, the power function model cannot be used to predict the later strength of concrete. (3) From Equation (3), the proposed model can be regarded as an improvement of the hyperbolic model by adding a term of $x_{2}/e^{t}$ into Equation (2). This improvement has two specific advantages. One is that the fitting accuracy for the super early age stage can be significantly improved by adding a new fitting parameter $x_{2}$. The other is that the predicted compressive strength by this new model also tends to be a constant over time. This is consistent with the real law of concrete strength development. Based on the above discussion, it has been shown that the proposed model can obtain more reasonable prediction results than the power function model and the hyperbolic model.

## 5. Conclusions

In this work, a new prediction model is proposed to analyze the variation of concrete compressive strength with age. Moreover, a robust regression analysis method is proposed to overcome the great discreteness in the measurement data to obtain the fitting parameters in the prediction model more accurately. Using the experimental data, the proposed prediction model is compared with the existing power function model and the hyperbolic function model. According to the comparative study based on experimental data, the main conclusions can be summarized as follows: (1) The power function model can be used to analyze the change of compressive strength of concrete at a super early age, but it can not be used to analyze the variation of the concrete compressive strength at a late age; (2) In general, the fitting accuracy of the hyperbolic model for the super early age concrete (within 3 days) is not ideal. Relatively, the hyperbolic model is more suitable for predicting the strength of concrete in the later stage; (3) The prediction model proposed in this work can be used not only to analyze the compressive strength of super early-age concrete, but also to predict the compressive strength of concrete after 28 days; (4) The proposed robust regression analysis method can effectively resist the great discreteness of the measured data and obtain the reliable fitting parameters in the mathematical model. It has been shown that the proposed prediction model may be an effective tool for predicting the early-age strength of concrete. The future research can be carried out around the following aspects: (1) The first aspect is to carry out more compressive strength experiments for the super early-age concrete to further verify and improve the proposed prediction model; (2) The second aspect is to study the applicability of the proposed model to the tensile strength and elastic modulus of the super early-age concrete; (3) The third aspect is to investigate the performance of the proposed model for analyzing the strength development law of other types of concrete, such as recycled concrete and high-strength concrete; (4) The fourth aspect is to apply the proposed model to the concrete strength prediction in real projects for better serving the construction practice.

## Figures and Tables

**Table 1:** The total fitting errors of the three prediction models.

Case	Power Function Model	Hyperbolic Model	Proposed Model
C30 (Natural curing, MPa)	0.0684	0.1351	0.0586
C30 (Standard curing, MPa)	0.0906	0.4738	0.2704
C40 (Natural curing, MPa)	0.2944	1.053	0.1751
C40 (Standard curing, MPa)	0.3599	1.4813	0.2735

**Table 2:** The total fitting standard deviations of the three prediction models.

Case	Power Function Model	Hyperbolic Model	Proposed Model
C30 (Natural curing, MPa)	0.011	0.0279	0.0085
C30 (Standard curing, MPa)	0.016	0.0814	0.0499
C40 (Natural curing, MPa)	0.0709	0.2266	0.0344
C40 (Standard curing, MPa)	0.0873	0.2685	0.0547

**Table 3:** Predicted compressive strengths of concrete with 28 days by the three models.

Case	Power Function Model	Hyperbolic Model	Proposed Model
C30 (Natural curing, MPa)	1783	44.3	40.5
C30 (Standard curing, MPa)	4955	137.7	52.9
C40 (Natural curing, MPa)	1226	104.1	50.4
C40 (Standard curing, MPa)	7451	−101.9	64.6

## Data Availability

Not applicable.
